# Another Test for Albumen in Urine

**Published:** 1883-05

**Authors:** 


Another Test for Albumen in Urine.—Dr. W. Roberts states in the *Lancet* that a saturated solution of salt in a fluid composed of one ounce of dilute sulphuric, nitric, or hydrochloric acid, with a pint of water, filtered, provides a test for albumen in urine as delicate as nitric acid. It should be applied in the same way, by trickling the solution down the sides of a test-tube containing the urine, held in a slanting direction.

The brine solution should be added in quantity equal to the urine, and if albumen be present it will be visible at the point of contact of the two liquids. This test is advantageous in the case of high-colored urines, which are made darker by nitric acid, and the brine solution is also less dangerous to carry in a pocket-case than the nitric acid.

